# Effect of *Panax notoginseng* Saponins and Major Anti-Obesity Components on Weight Loss

**DOI:** 10.3389/fphar.2020.601751

**Published:** 2021-03-25

**Authors:** Xuelian Zhang, Bin Zhang, Chenyang Zhang, Guibo Sun, Xiaobo Sun

**Affiliations:** ^1^ Institute of Medicinal Plant Development, Peking Union Medical College and Chinese Academy of Medical Sciences, Beijing, China; ^2^ Key Laboratory of Bioactive Substances and Resources Utilization of Chinese Herbal Medicine, Ministry of Education, Beijing, China; ^3^ Beijing Key Laboratory of Innovative Drug Discovery of Traditional Chinese Medicine (Natural Medicine) and Translational Medicine, Beijing, China; ^4^ Key Laboratory of Efficacy Evaluation of Chinese Medicine Against Glyeolipid Metabolism Disorder Disease, State Administration of Traditional Chinese Medicine, Beijing, China

**Keywords:** anti-obesity, adipogenesis, lipolysis, browning of white adipose tissue, insulin sensitivity

## Abstract

The prevalence of individuals who are overweight or obese is rising rapidly globally. Currently, majority of drugs used to treat obesity are ineffective or are accompanied by obvious side effects; hence, the options are very limited. Therefore, it is necessary to find more effective and safer anti-obesity drugs. It has been proven *in vivo* and *in vitro* that the active ingredient notoginsenosides isolated from traditional Chinese medicine *Panax notoginseng* (Burk.) F. H. Chen exhibits anti-obesity effects. Notoginsenosides can treat obesity by reducing lipid synthesis, inhibiting adipogenesis, promoting white adipose tissue browning, increasing energy consumption, and improving insulin sensitivity. Although notoginsenosides are potential drugs for the treatment of obesity, their effects and mechanisms have not been analyzed in depth. In this review, the anti-obesity potential and mechanism of action of notoginsenosides were analyzed; thus laying emphasis on the timely prevention and treatment of obesity.

## Introduction

Individuals with excess fat accumulation that can impair health are called overweight or obese. According to the World Health Organization, an adult with a body mass index (BMI) ≥25 is overweight and ≥30 is obese. The global prevalence of obesity almost tripled between 1975 and 2016, with 39% of adults classified as overweight and 13% as obese (Collaboration, 2017). Obesity often increases the risk of lifestyle diseases, such as cardiovascular disease, diabetes, and cancer. Although a healthier lifestyle, such as healthy food and regular exercise can prevent obesity and promote weight loss; but, in the long run, lifestyle, environmental, and genetic factors can easily lead to obesity again (Dulloo and Montani, 2015). Therefore, drug therapy combined with exercise and diet management is extremely beneficial for individuals who struggle with weight loss. However, currently, there are very few drugs available for obesity treatment. Currently, only Orlistat, Lorcaserin, and Qsymia (Fentiemine/Topiramate sustained-release tablets) have been approved by the FDA for use for long-term weight control. Orlistat is an intestinal lipase inhibitor; by inhibiting the hydrolysis of triglycerides, Orlistat reduces the absorption of fat from food to achieve weight loss. However, long-term use of the drug can lead to deficiency of fat-soluble vitamins and cause gastrointestinal disorders (Filippatos et al., 2008; Halpern and Halpern, 2015). Lorcaserin, which can reduce appetite, acts on the 5-HT2C receptor as a sympathetic nerve agent, but its long-term use has put individuals at risk for valvular heart disease and cancer (Greenway et al., 2016). Qsymia is a central nervous system weight-loss drug that has precipitated adverse effects, such as headache, insomnia, constipation, and dizziness (Siebenhofer et al., 2016; Halpern and Mancini, 2017). In addition, many weight-loss medications have been withdrawn due to strong side effects, such as Flaviprolamine (heart disease), Amirese (obstructive pulmonary hypertension), Phentamine (insomnia, fatal pulmonary hypertension), and Rimonabant (psychiatric reactions, depression and anxiety, suicide risk) (Cheung et al., 2013). Therefore, the development of safe and effective new anti-obesity drugs has great clinical significance and economic value.

**FIGURE 1:**
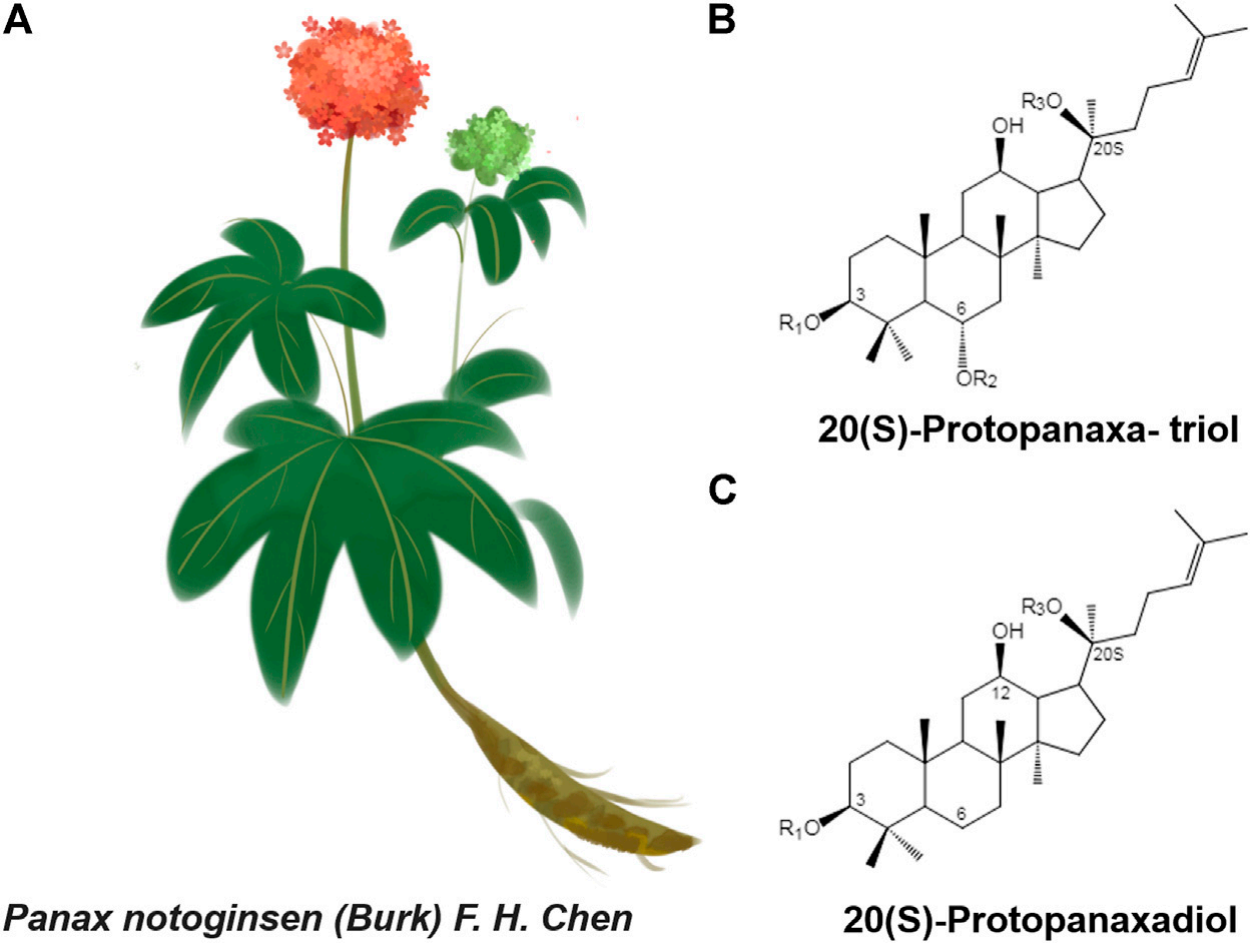
**(A)** *Panax notoginseng* F. H. Chen **(B and C)** Structure skeletons of 20(S)-protopanaxatriol and 20(S)-protopanaxadiol that exist in raw and processed *Panax notoginseng.*

*Panax notoginseng* (Burk.) F. H. Chen (Sanqi in Chinese) is a valuable traditional Chinese medicine, belonging to the genus Araliaceae, that was first recorded in Shennong Ben Cao *Jing.* Its dried roots promote blood circulation, stop bleeding, reduce swelling, and relieve pain. With developments in traditional medicine, a more systematic study of *Panax notoginseng* has been carried out using modern physics, chemical technology, and modern medical theories. More than 80 types of monomeric saponins, flavonoids, and notoginseng phytoconstituents with unique pharmacological activities were isolated from different parts of *P. notoginseng*, among which *P. notoginseng* saponins (PNS) are the main active components. The PNS are divided into two groups, 20(S)-protopanaxatriol or 20(S)-protopanaxadiol (Qiao et al., 2018) (Figure 1). These components have a variety of pharmacological activities, such as anti-oxidation (Hu et al., 2018; Hu et al., 2019), anti-depression (Xiang et al., 2011; Cui et al., 2012; Zhang et al., 2018), treatment of cardiovascular diseases (Zhou et al., 2018; Liu X. W. et al., 2019) and treatment of diabetes (Guo et al., 2019; Wang et al., 2019).

Recent studies have found that PNS or monomer notoginsenosides have regulatory effects on obesity and lipid metabolism in addition to the treatment of type 2 diabetes and atherosclerosis (Fan et al., 2012; Peng et al., 2019). When the type 2 diabetes model KK-Ay mice were treated with PNS, it was found that PNS decreased the fasting blood glucose level of diabetic mice accompanied with significant weight loss (Yang et al., 2009). Further studies showed that PNS could downregulate SREBP1, SCD1, and FAS through the AMPK signaling pathway, inhibit adipogenesis, and reduce white adipose tissue weight, thereby reducing the weight of diabetic mice (Wang et al., 2020). In high-fat diet-induced atherosclerosis models, treatment with the PNS/monomer notoginsenosides significantly reduced lipid levels (Jia et al., 2010; Fan et al., 2012; Liu C. et al., 2019). In addition, PNS can inhibit lipase protein expression in non-alcoholic fatty liver disease (NAFLD), reduce lipolysis in white adipose tissue, and thus alleviate lipid accumulation in the liver (Ding et al., 2015). In addition, PNS, notoginsenosides R1, ginsenoside Rg1, and ginsenoside Rb1 can significantly reduce the content of SREBP2 and HMG-CoA, directly inhibit the synthesis of cholesterol, and protect the liver (Chen et al., 2016).

However, so far, pharmacological studies of PNS and monomer notoginsenosides have mostly focused on cardiovascular system diseases, central nervous system diseases, and anticancer activities. There is a lack of systematic evaluation of their role in regulating lipid metabolism and their anti-obesity effects. This review summarizes the anti-obesity effects and mechanisms of PNS, provides research potential for the clinical treatment of obesity and the development of safe and effective anti-obesity drugs.

## Effects on Adipogenesis and Lipolysis

### Adipogenesis

Due to the long-term imbalance of energy intake and consumption by the body, excess energy is stored in white adipocytes in the form of triglycerides (Kopelman, 2000). This process is accompanied by the proliferation and differentiation of preadipocytes and cell hypertrophy, resulting from increased lipid storage that eventually leads to the proliferation of adipose tissue volume, presenting as obesity (Arner and Spalding, 2010). In mammalian cells, peroxisome proliferator-activated receptor (PPAR) and CCAAT/enhancer binding protein (C/EBP) are considered to be key early regulators of adipogenesis (Wu et al., 1999; Rosen et al., 2000; Lefterova and Lazar, 2009), whereas SREBP1 and cAMP response element binding protein (CREB) regulate adipose tissue differentiation and metabolism by influencing PPAR (Kim and Spiegelman, 1996; Fajas et al., 1999; Reusch et al., 2000). Additionally, proteins, such as glucose transporter 4 (GLUT4), lipoprotein lipase (LPL), SCD, and FAS, are also involved in adipogenesis and lipid storage (Moseti et al., 2016).

The experimental results (Table 1) of *in vivo* and *in vitro* studies showed that many kinds of notoginseng monomer saponins, such as ginsenosides RC, Rg3, and Rg2, can inhibit the proliferation and differentiation of adipocytes, reduce the number of adipocytes, and inhibit the accumulation of lipids in adipocytes by reducing the expression levels of PPARγ, C/EBPs, SREBP, and FAS, to achieve anti-obesity effects. The combination of various saponins, such as ginsenosides Rb1, Rg1, and notoginsenosides R1, can also reduce the synthesis of lipids and cholesterol through SREBP-1C, acetyl-CoA carboxylase (ACC), acetyl-coa synthetase (ACS), and other proteins (Xie et al., 2015). In addition, the AMPK pathway acts as a central regulator of cellular energy sensors, organelle biogenesis, cell metabolism, cell proliferation, and differentiation (Hardie, 2011). Current studies have shown that activation of AMPK signaling increases energy consumption and reduces lipid accumulation and adipogenesis (Fernandez-Veledo et al., 2013; Desjardins and Steinberg, 2018). Ginsenosides Rb1, Rg3, CK, and Rg1, can improve insulin sensitivity and inhibit the occurrence of obesity by activating the AMPK pathway. The *in vitro* and *in vivo* anti-obesity effect and mechanism of some notoginsenosides have been discussed at present as some drugs are still lacking *in vivo* experimental verification, and the mechanism of action needs to be further explored.

**TABLE 1:** Effect of notoginsenosides on adipogenesis.

Durg	Type	Effect	Mechanism	References
Ginsenoside Rb1	*In vivo*	Anti-obesity, improved insulin sensitivity	Elevated activation of hepatic AMP-activated protein kinase (AMPK) and phosphorylated acetyl-CoA carboxylase	Shen et al. (2013)
Ginsenoside Rb2	*In vitro*	Decreased TAG levels	Stimulated the expression of SREBP and leptin mRNA	Kim E. J. et al. (2009)
Ginsenoside Rc	*In vitro*	Decreased the number of adipocytes, reduced lipid accumulation in maturing preadipocytes	Down-regulated the expression of PPARγ and C/EBPα	Yang and Kim (2015)
Ginsenoside Rg3	*In vitro* and *vivo*	Reduced serum levels of triglyceride, total cholesterol, and LDL-cholesterol; reduced lipid accumulation in adipocytes and suppressed adipogenesis; reduced epididymal white adipose tissue size; improved insulin sensitivity	Increased AMPK activation and suppressed adipogenesis by decreasing the mRNA expression of C/EBPα, PPARγ, SREBP1, Pgc-1α, FAS, AP2, and SIRT1 and by increasing that of CPT1 and HSL.	Hwang et al. (2009); Lee et al. (2012); Lee et al. (2017); Zhang et al. (2017); Lee H. S. et al. (2019); Kim et al. (2020)
Ginsenoside F2	*In vitro*	Reduced lipid accumulation	Reduced the gene expression of PPARγ and perilipin	Siraj et al. (2015)
Ginsenoside CK	*In vitro*	Enhanced glucose uptake; inhibited triglyceride accumulation	Activation of AMPK and PI3K signaling pathways; induced GLUT4 expression at both the mRNA and protein levels	Huang et al. (2010)
Ginsenoside Rg1	*In vitro* and *viv*o	Decreased body weight, total cholesterol, and total triglyceride levels; inhibited lipogenesis, and decreased intracellular lipid content, adipocyte size, and adipose weight	Induced AMPK activation; increased CHOP10 and reduced the C/EBPβ transcriptional activity; reduced fat and cholesterol anabolism genes such as SREBP-1c, ACC, ATP-CL, ACS; promoted the expression of PPAR-α, CPT1A, CPT2, and CYP-7A	Xie et al. (2015); Koh et al. (2017); Liu et al. (2018); Lee J. H. et al. (2019); Hou et al. (2020)
Ginsenoside Rg2	*In vitro* and *vivo*	Inhibited adipocyte differentiation and decreased body weight, reversed hepatic steatosis, and improved glucose tolerance and insulin sensitivity	Induced activation of AMPK and SIRT1 signaling pathway; decreased the expression levels of PPARγ, C/EBPα, and SREBP1-c, and then regulated target genes such as ACC and FAS	Liu H. et al. (2019); Cheng et al. (2020)
Ginsenoside Rh1	*In vitro* and *vivo*	Suppressed body and epididymal fat weight gains and plasma triglyceride level; inhibited adipogenesis	Decreased the expressions of PPAR-γ, C/EBP-α, FAS, and FABP	Gu et al. (2013)

### Lipolysis

In obesity, excess circulating fatty acids in plasma may accumulate ectopically in insulin-sensitive tissues and impair insulin action. Increased basal lipolysis may also change the secretion status of adipose tissues, affect the insulin sensitivity of the whole body, and exacerbate the inflammation of adipose tissue (Morigny et al., 2016). Therefore, in addition to inhibiting the proliferation and differentiation of adipocytes, regulating adipolysis is also crucial for the management of obesity.

In the experimental studies reported so far, the regulation of notoginsenosides on adipolysis involves several different mechanisms; Ginsenosides, Rg1, Rg2, and Rh1, regulate PPAR, C/EBP, and AMPK signaling pathways, while inhibiting adipogenesis, reduce lipid synthesis and inhibit lipolysis, thereby reducing circulating blood lipid levels and improving insulin sensitivity (Masuno et al., 1996; Lee K. et al., 2019). Interestingly, Hyun Sook Lee et al. found that ginsenoside Rg3 can directly increase the expression of lipolysis-related genes in the white adipose of obese mice that were fed a high-fat diet and promote lipolysis by upregulating crucial genes *CPT1* and *HSL* (Rupasinghe et al., 2016; Dankel et al., 2019; Lee H. S. et al., 2019). However, there are also reports on Ginsenoside Rb1-induced beta three adrenergic receptor-dependent lipolysis and thermogenesis. Ginsenoside Rb1 treatment can increase the protein expression of lipase and thermogenic factor UCP1, increase the level of lipolysis, and reduce the size of adipocytes, and meanwhile increase the thermogenic capacity of obese mice, thereby consuming free fatty acids (Lim et al., 2019). In addition, 20(S)-ginsenoside Rg3 can exert a similar effect to Orlistat by regulating pancreatic lipase. Experiments have shown that 20(S)-ginsenoside Rg3, which is a potential drug for the treatment of obesity, can inhibit pancreatic lipase activity, reduce the decomposition and absorption of lipids in food, and inhibit lipid accumulation during the differentiation of 3T3-L1 adipogenic cells through AMPK and PPAR-signaling pathways. (Fei et al., 2016).

## Effects on Body Energy Consumption

### Promote White Adipose Tissue Browning

There are two main types of adipocytes in mammals: white adipose tissue (WAT), which is the major tissue for energy storage, and brown adipose tissue (BAT), which is the major tissue for energy consumption. WAT converts the excess energy of the body into triglycerides and stores them in cells, whereas BAT contains a large number of mitochondria, among which high levels of UCP1 consume bioenergy and emit energy in non-tremor thermogenesis to maintain human body temperature and energy consumption (Contreras et al., 2016; Lee J. H. et al., 2019). More recently, a third type of adipocyte, called beige adipocytes, has been identified in WAT that is similar to classic brown adipocytes but with comparatively higher levels of UCP1 (Giordano et al., 2014). Biogenesis of beige adipocytes in WAT can be induced by cold exposure and drug or hormone stimulation (de Jong et al., 2017). In the past few years, the development and transcriptional regulation of beige fat has received much attention. It has been found that genetically and pharmacologically inducing beige adipocytes can protect mice from obesity and insulin resistance induced by a high-fat diet, as well as effectively increase energy expenditure and improve metabolic disorders. Recent studies have also identified several major transcriptional regulators of the development and function of beige adipocytes, including PPARs, PGC1, FOXC2, and, PRDM16 (Su et al., 2018; Lizcano, 2019).

At present, many studies have shown that ginsenoside Rb1 induces browning of adipocytes through AMPK-mediated pathways thereby exerting anti-obesity effects (Mu et al., 2015; Park S. J. et al., 2019; Lim et al., 2019). *In vitro*, 10 μM ginsenoside Rb1 treatment increased glucose uptake in 3T3-L1 cells and promoted mRNA expression of brown fat marker proteins UCP-1, PGC-1α, and PRDM16. In addition, ginsenoside Rb1 also increased PPARγ expression (Mu et al., 2015; Park S. J. et al., 2019), and browning was eliminated with PPARγ antagonist GW9692 (Mu et al., 2015). *In vivo*, db/db mice were intraperitoneally injected with ginsenoside Rb1, the amount of WAT in the groin decreased, and respiration and heat production increased. After pre-treatment with β3AR antagonist L748337, ginsenoside Rb1 lost its ability to promote browning and thermogenesis (Lim et al., 2019). In addition, other studies have found that ginsenosides Rb2, Rg1, and Rg3 induced AMPK phosphorylation, stimulated the expression of UCP1, and increased thermogenesis and mitochondrial gene expression to induce BAT activation and white adipocyte browning (Lee K. et al., 2019; Hong et al., 2019; Kim et al., 2020). Ginsenoside Rd enhances thermal gene expression in brown adipose tissue through the PKA signaling pathway and increases browning of white adipose tissue induced by cold exposure (Yao et al., 2020).

As shown in Table 1, many panax notoginseng monomer saponins can affect body adipogenesis by regulating PPARs and AMPK. PPARs are a group of nuclear transcription factors; there are three types of PPAR: PPARα, δ, andγ. PPARγ is a core transcription factor required for white adipogenesis (Rosen and Spiegelman, 2014). When PPARγ binds to PRDM16, PPARγ stimulates the expression of selective genes in brown and beige adipocytes and inhibits white adipocyte specific genes (Ohno et al., 2012; Qiang et al., 2012). PPARα and PPAR δ stimulate fatty acid oxidation and mitochondrial respiration, promoting white adipose tissue browning (Wang et al., 2003; Hondares et al., 2011; Mottillo et al., 2012; Barquissau et al., 2016). In addition, studies have shown that activation of AMPK can increase the activity and energy expenditure of BAT and beige adipocytes and targeting AMPK may have therapeutic potential for treating obesity and related diseases (Fernandez-Marcos and Auwerx, 2011; van Dam et al., 2015). Therefore, in addition to the observed inhibition of adipogenesis and weight loss by AMPK and PPARs, we can also explore whether this component can resist obesity by promoting browning of white adipose tissue and increasing body energy consumption.

### Mitochondrial Protective Effect

Mitochondria are essential organelles for energy metabolism. Adipocyte mitochondria play a substantial regulatory role between whole-body energy balance, muscle and adipose tissue differentiation, and insulin sensitivity and glucose metabolism (Bhatti et al., 2017). Various studies have shown that mitochondrial function and biogenesis are impaired in type 2 diabetes, obesity, and insulin-resistant adipose tissue (Bhatti et al., 2017; Dai and Jiang, 2019). The main function of mitochondria in adipocytes is to produce ATP to support a variety of key metabolic pathways for lipid clearance, including triglyceride synthesis, glucometosis, and fatty acid oxidation. In addition, in response to cold exposure, drugs or adrenal hormones, activation of mitochondria in brown and beige adipocytes accelerates energy consumption by increasing the expression of mitochondrial UCP1 (Harms and Seale, 2013). Fatty acid oxidation and reduced energy consumption, caused by mitochondrial dysfunction in the brown adipocytes of individuals with obesity and metabolic diseases, indicate the role of mitochondrial function in anti-obesity effects (Bournat and Brown, 2010).

Excessive reactive oxygen species (ROS) production in adipose tissue of obese mice and significantly decreased mitochondrial mtDNA and respiratory protein expression, resulting in mitochondrial dysfunction (Furukawa et al., 2004; Sparks et al., 2005). Ginsenosides Rb2, F1, and Rc enhanced the deacetylation activity of SIRT1 and inhibited the formation of intracellular ROS. In addition, by increasing mitochondrial DNA content, cell oxygen consumption was restored, and mitochondrial damage induced by oxidative stress was reduced, showing the protective effect of mitochondrial function (Wang et al., 2016). Ginsenosides Rd and Re reduce oxidative stress, improve mitochondrial integrity and function, and inhibit intracellular ROS production and lipid peroxidation caused by rotenone (Gonzalez-Burgos et al., 2017). Ginsenosides Rg3 and Rg1 can regulate mitochondrial autophagy and biogenesis by activating the AMPK signaling pathway, thus improving mitochondrial dysfunction (Xing et al., 2017; Lee K. et al., 2019). Thus, notoginsenosides may protect mitochondrial function by regulating mitochondrial energy metabolism, oxidative stress, biogenesis, autophagy, and enhance energy consumption in obese patients. In addition, it has been reported that inhibition of fatty acid oxidation and maintenance of mitochondrial energy metabolism are essential for the survival of brown and beige adipocytes during dormancy (Kutyavin and Chawla, 2019). Therefore, the mitochondrial protective effect of notoginsenosides may affect the thermogenic function maintenance of BAT and beige adipocytes in a thermally neutral environment, which needs further experimental investigation.

## Effects on Insulin Sensitivity

### Obesity and Insulin Resistance

It has been shown that obesity-induced adipose tissue metabolic disorders cause primary insulin resistance in insulin-sensitive tissues (Smith and Kahn, 2016; Czech, 2017). Lipid overload and lipid toxicity caused by obesity affect insulin sensitivity of various organs by interfering with the insulin signal transduction pathway (Chen et al., 2017; Engin, 2017). In contrast, fatty factors secreted by adipose tissue, such as monocyte chemoattractant protein-1/chemokine (C-C motif) ligand-2 (McP-1/CCL2) and tumor necrosis factor-α (TNFα), regulate inflammatory responses in adipose tissue (Sartipy and Loskutoff, 2003). McP-1/CCL2 acts as a chemoattractant that increases the macrophage content in adipose tissue in obese patients and causes chronic low-grade inflammation in adipose tissue (Weisberg et al., 2003; Curat et al., 2004). The chronic inflammatory state of obesity is associated with excessive production of TNFα, which downregulates PPARγ expression (Zhang et al., 1996). The downregulation of PPARγ protein expression leads to a decrease in adipogenesis, and the storage capacity of triglycerides in adipocytes is impaired, which increases the level of free FFA, resulting in a vicious circle (Abdullahi and Jeschke, 2016). In addition, inflammation may cause insulin resistance through the direct action of TNFα on muscle insulin signaling. In addition, the normal secretion of adipogenic factors, such as leptin and adiponectin, is also affected by adipose tissue metabolic disorders (Berg et al., 2002). Improving insulin resistance is important in the treatment of obesity and obesity-related lifetsyle disorders.

Ginsenoside Rg3 was found to improve the pathological changes caused by obesity by downregulating STAT5-PPAR. Rg3-treated 3T3-L1 cells showed reduced lipid accumulation and total TG levels, and alleviated obesity-induced insulin resistance and lipid toxicity (Lee et al., 2017). By measuring the expression level of apoptosis-related protein and TUNEL staining, it was found that ginsenoside Rb2 improved the insulin resistance and apoptosis of 3T3-L1 adipocytes induced by TNF-α (Lin et al., 2020), and ginsenoside Rd reduced the BAX/BCL2 ratio and directly reduced the apoptosis of islet cells (Kaviani et al., 2019). In addition, it has been found that ginsenoside Rg3 can improve insulin signaling in obese patients by stimulating the expression of IRS-1 and GLUT4 (Kim M. et al., 2009), and ginsenosides Rb1, Rg1, Rg3, and Rh2 can enhance glucose-stimulated insulin secretion in islet cells (Park et al., 2008; Yuan et al., 2012). It can be seen that notoginsenosides can improve insulin sensitivity of the body by alleviating lipid toxicity, protecting adipocytes and islet cells, and enhancing insulin signal transduction. However, interestingly, Reeds et al. found that oral administration of ginseng and ginsenoside Re did not improve impaired glucose tolerance or obesity, and there were no significant changes in cell function or insulin sensitivity in obese subjects, which may be related to the low systemic bioavailability of saponins (Reeds et al., 2011). This question needs to be answered with quality evidence in subsequent experimental studies to confirm the ginsenoside efficacy.

### Glucose Metabolism

Adipose tissue plays an important role in the control of systemic glucose homeostasis in both normal and diseased states. Insulin resistance associated with obesity indicates a decrease in the body’s ability to activate the insulin signaling pathway, which stimulates glucose uptake and metabolism (Villalobos-Labra et al., 2019). In the adipocytes of insulin-resistant obese patients, reduced levels of the insulin-regulated glucose transporter GLUT4 trigger hyperglycaemia (Brannmark et al., 2013) thus activating oxidative stress, inflammation, and endoplasmic reticulum stress responses (Mozzini et al., 2015; Mota et al., 2016).

**FIGURE 2:**
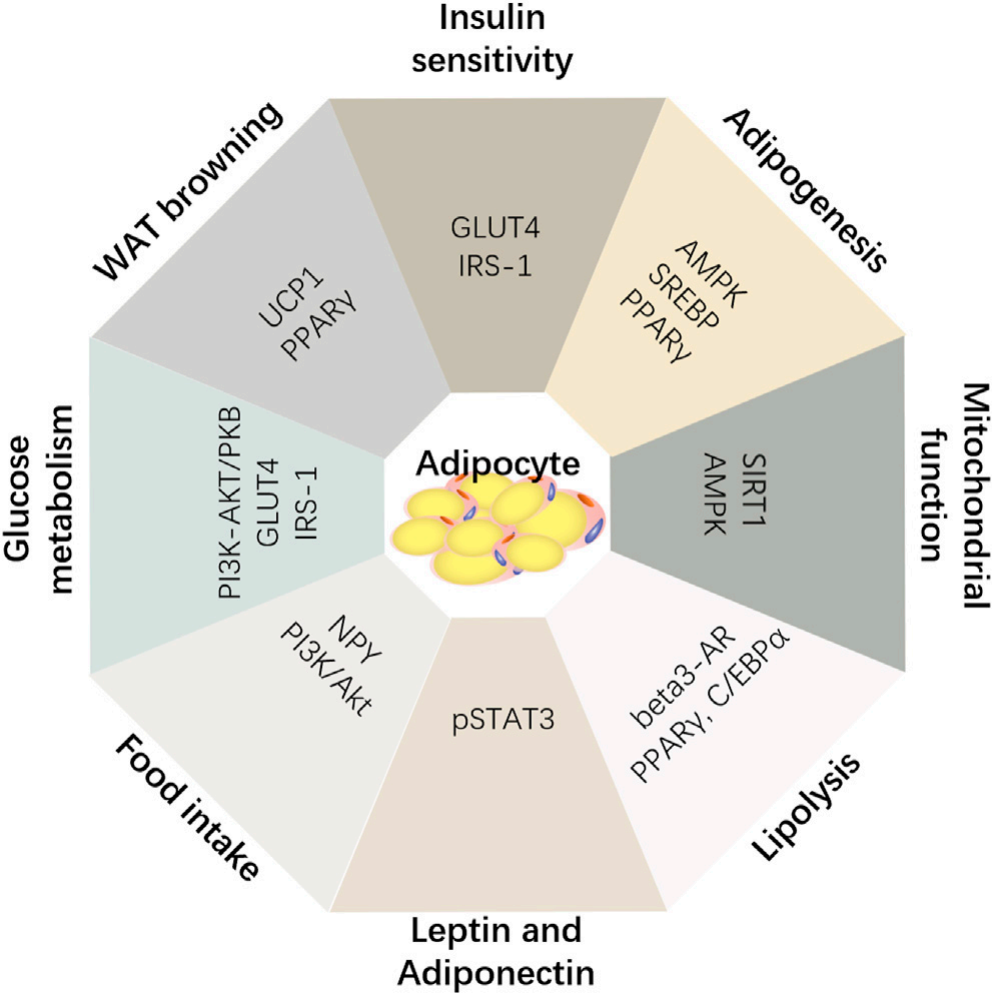
Summary of anti-obesity mechanism of notoginsenosides.

Ginsenosides Rb2 and Rg3 were found to increase glucose uptake by the IRS-1-PI3K-Akt/PKB pathway in 3T3-L1 adipocytes (Lee et al., 2011; Dai et al., 2018). Ginsenosides Rb1, Re, CK, and Rg1 promote the absorption and utilization of glucose in adipocytes, liver, and muscle tissues and improve insulin resistance by activating AMPK pathways and increasing GLUT4 mRNA and protein levels (Huang et al., 2010; Shang et al., 2014; Li et al., 2018). Although adipose tissue absorbs less glucose than skeletal muscle, it accounts for only about 10% of the glucose load at mealtime. However, it has recently been found that GLUT4 expression and glucose metabolism in adipose cells can affect substrate metabolism and adipogenesis by changing endocrine functions, thus improving lipid metabolism disorders caused by obesity (Figure 2) (Semirale et al., 2011; Smith and Kahn, 2016).

## Conclusion

Obesity has become an epidemic worldwide due to genetics, or poor lifestyle habits, such as fatty diet and less exercise. According to statistics, obesity-related diabetes, cardiovascular disease, and cancer have shortened the life span of obese patients by 4–7 years (Bray et al., 2018). At present, most of the drugs and strategies to manage obesity have obvious side effects or are not effective. Moreover, obesity is can cause a variety of pathological changes, including hyperlipidemia, chronic inflammation, adipocyte dysfunction, glucose and lipid metabolism disorder, mitochondrial injury and insulin resistance, and other multi-factor interactions, making the treatment of obesity more difficult. For example, in response to increased lipid levels, mitochondrial function is downregulated and the expression of oxidative phosphorylation genes is reduced (Richardson et al., 2005; Koves et al., 2008). However, reduced mitochondrial function results in impaired ability to consume fatty acids through oxidative metabolism that may further exacerbate lipid toxicity and glucose lipid metabolism disorders, secondary inflammation, and insulin resistance (Morino et al., 2006).

*P. notoginseng* contains a variety of phytoconstituents, including notoginsenosides, ginsenosides, quercetin, and ginseng polysaccharides, that have been used as a rare traditional Chinese medicine for many centuries. There is much evidence to support the effect of notoginsenosides in the management of obesity and weight loss. As mentioned above, notoginseng saponins regulate adipogenesis and lipolysis through signaling pathways, such as AMPK and PPAR, reducing adipose tissue volume while avoiding the generation of lipid toxicity. In addition, notoginsenosides also alleviate the mitochondrial metabolic disorders associated with obesity and protect mitochondrial function through antioxidant stress. They promote browning of white adipose tissue by PPARs and PGC-1Α, such that energy storage tissue can be transformed into energy consumption tissue, and increase the body's energy consumption to resist obesity. Furthermore, notoginsenosides can also improve glucose and lipid metabolism disorders caused by obesity. By improving the expression of GLUT4 protein and improving the insulin sensitivity of all organs and tissues, and promoting the uptake and utilization of glucose and free lipids in all organs, obesity can be managed from various aspects.

In addition, it has been reported that ginsenosides treat obesity in other ways. Ginsenoside Rb1 decreased pro-inflammatory cytokines, such as TNF-α, IL-6, and NF-κB induced by a high-fat diet, and restored leptin signaling in the hypothalamus and pSTAT3 in high-fat mice (Wu et al., 2014; Wu et al., 2018). In addition, ginsenoside Rb1 can improve insulin sensitivity in obese and diabetic db/db mice by upregulating plasma adiponectin levels, reducing liver fat accumulation, and inhibiting adipocyte lipolysis (Yu et al., 2015). Interestingly, acute intraperitoneal injection of Rb1 activates the PI3K/Akt signaling pathway through the expression of c-FOS in brain regions involved in energy homeostasis, and inhibits the expression of NPY gene in the hypothalamus, thereby reducing appetite and food intake, body weight, and body fat content and increasing energy consumption (Xiong et al., 2010; Park H. J. et al., 2019). Xu et al. also found that PNS-induced regulation of intestinal flora in DIO mice can increase BAT thermogenesis and beige adipocyte reconstruction by activating the leptin-AMPK/STAT3 signaling pathway thus promoting energy consumption (Xu et al., 2020).

At present, it is difficult to achieve significant therapeutic effects in the treatment of complex diseases using a single target strategy. Although the efficacy of some notoginsenosides needs to be further confirmed, and the detailed mechanism of notoginsenosides in the treatment of obesity needs to be further elucidated, notoginsenosides show the possibility of resisting obesity in many ways, providing new ideas and methods for the multi-target treatment of such complex diseases. In addition, the oral utilization rates of different *P. notoginseng* saponins were significantly different; oral utilization of PPD ginsenoside was significantly higher than that of PPT ginsenoside. However, in general, PNS has poor absorption, long elimination half-life, and low bioavailability. PNS is metabolized by bacteria and enzymes in the gastrointestinal tract with many glycosides (Han et al., 2006; Liu et al., 2009). At present, there is insufficient evidence to support the clinical application of notoginsenosides in the treatment of obesity. Clinical trials of the safety and efficacy of these compounds are needed to verify the effects of saponins observed *in vitro* and in animal models.

## References

[B1] Abdullahi A. Jeschke M. G. (2016). White adipose tissue browning: a double-edged sword. Trends Endocrinol. Metab. 27 (8), 542–552. 10.1016/j.tem.2016.06.006 27397607 PMC5234861

[Bitem] AnonymousAuthor (2012). LiverTox: clinical and research information on drug-induced liver injury. Bethesda, MD: National Institute of Diabetes and Digestive and Kidney Diseases.

[B2] Arner P. Spalding K. L. (2010). Fat cell turnover in humans. Biochem. Biophys. Res. Commun. 396 (1), 101–104. 10.1016/j.bbrc.2010.02.165 20494119

[B3] Barquissau V. Beuzelin D. Pisani D. F. Beranger G. E. Mairal A. Montagner A. (2016). White-to-brite conversion in human adipocytes promotes metabolic reprogramming towards fatty acid anabolic and catabolic pathways. Mol. Metab. 5 (5), 352–365. 10.1016/j.molmet.2016.03.002 27110487 PMC4837301

[B4] Berg A. H. Combs T. P. Scherer P. E. (2002). ACRP30/adiponectin: an adipokine regulating glucose and lipid metabolism. Trends Endocrinol. Metab. 13 (2), 84–89. 10.1016/s1043-2760(01)00524-0 11854024

[B5] Bhatti J. S. Bhatti G. K. Reddy P. H. (2017). Mitochondrial dysfunction and oxidative stress in metabolic disorders - a step towards mitochondria based therapeutic strategies. Biochim. Biophys. Acta 1863 (5), 1066–1077. 10.1016/j.bbadis.2016.11.010 PMC5423868 27836629

[B6] Bournat J. C. Brown C. W. (2010). Mitochondrial dysfunction in obesity. Curr. Opin. Endocrinol. Diabetes Obes. 17 (5), 446–452. 10.1097/MED.0b013e32833c3026 20585248 PMC5001554

[B7] Brännmark C. Nyman E. Fagerholm S. Bergenholm L. Ekstrand E. M. Cedersund G. (2013). Insulin signaling in type 2 diabetes: experimental and modeling analyses reveal mechanisms of insulin resistance in human adipocytes. J. Biol. Chem. 288 (14), 9867–9880. 10.1074/jbc.M112.432062 23400783 PMC3617287

[B8] Bray G. A. Heisel W. E. Afshin A. Jensen M. D. Dietz W. H. Long M. (2018). The science of obesity management: an endocrine society scientific statement. Endocr. Rev. 39 (2), 79–132. 10.1210/er.2017-00253 29518206 PMC5888222

[B9] Chen X. J. Liu W. J. Wen M. L. Liang H. Wu S. M. Zhu Y. Z. (2017). Ameliorative effects of compound K and ginsenoside Rh1 on non-alcoholic fatty liver disease in rats. Sci. Rep. 7, 41144. 10.1038/srep41144 28106137 PMC5247696

[B10] Chen Z. Li C. Yang C. Zhao R. Mao X. Yu J. (2016). Lipid regulation effects of raw and processed notoginseng radix et rhizome on steatotic hepatocyte L02 cell. Biomed. Res. Int. 2016, 2919034. 10.1155/2016/2919034 27642594 PMC5013208

[B11] Cheng B. Gao W. Wu X. Zheng M. Yu Y. Song C. (2020). Ginsenoside Rg2 ameliorates high-fat diet-induced metabolic disease through SIRT1. J. Agric. Food Chem. 68 (14), 4215–4226. 10.1021/acs.jafc.0c00833 32181656

[B12] Cheung B. M. Cheung T. T. Samaranayake N. R. (2013). Safety of antiobesity drugs. Ther. Adv. Drug Saf. 4 (4), 171–181. 10.1177/2042098613489721 25114779 PMC4125319

[B13] Collaboration N. C. D. R. F. (2017). Worldwide trends in body-mass index, underweight, overweight, and obesity from 1975 to 2016: a pooled analysis of 2416 population-based measurement studies in 128·9 million children, adolescents, and adults. Lancet 390 (10113), 2627–2642. 10.1016/S0140-6736(17)32129-3 29029897 PMC5735219

[B14] Contreras C. Nogueiras R. Diéguez C. Medina-Gómez G. López M. (2016). Hypothalamus and thermogenesis: heating the BAT, browning the WAT. Mol. Cell. Endocrinol. 438, 107–115. 10.1016/j.mce.2016.08.002 27498420

[B15] Cui J. Jiang L. Xiang H. (2012). Ginsenoside Rb3 exerts antidepressant-like effects in several animal models. J. Psychopharmacol. 26 (5), 697–713. 10.1177/0269881111415735 21948936

[B16] Curat C. A. Miranville A. Sengenès C. Diehl M. Tonus C. Busse R. (2004). From blood monocytes to adipose tissue-resident macrophages: induction of diapedesis by human mature adipocytes. Diabetes 53 (5), 1285–1292. 10.2337/diabetes.53.5.1285 15111498

[B17] Czech M. P. (2017). Insulin action and resistance in obesity and type 2 diabetes. Nat. Med. 23 (7), 804–814. 10.1038/nm.4350 28697184 PMC6048953

[B18] Dai S. Hong Y. Xu J. Lin Y. Si Q. Gu X. (2018). Ginsenoside Rb2 promotes glucose metabolism and attenuates fat accumulation via AKT-dependent mechanisms. Biomed. Pharmacother. 100, 93–100. 10.1016/j.biopha.2018.01.111 29425748

[B19] Dai W. Jiang L. (2019). Dysregulated mitochondrial dynamics and metabolism in obesity, diabetes, and cancer. Front. Endocrinol. 10, 570. 10.3389/fendo.2019.00570 PMC6734166 31551926

[B20] Dankel S. N. Røst T. H. Kulyté A. Fandalyuk Z. Skurk T. Hauner H. (2019). The Rho GTPase RND3 regulates adipocyte lipolysis. Metabolism 101, 153999. 10.1016/j.metabol.2019.153999 31672447

[B21] de Jong J. M. A. Wouters R. T. F. Boulet N. Cannon B. Nedergaard J. Petrovic N. (2017). The β3-adrenergic receptor is dispensable for browning of adipose tissues. Am. J. Physiol. Endocrinol. Metab. 312 (6), E508–E518. 10.1152/ajpendo.00437.2016 28223294

[B22] Desjardins E. M. Steinberg G. R. (2018). Emerging role of AMPK in Brown and beige adipose tissue (BAT): implications for obesity, insulin resistance, and type 2 diabetes. Curr. Diabetes Rep. 18 (10), 80. 10.1007/s11892-018-1049-6 30120579

[B23] Ding R. B. Tian K. Cao Y. W. Bao J. L. Wang M. He C. (2015). Protective effect of panax notoginseng saponins on acute ethanol-induced liver injury is associated with ameliorating hepatic lipid accumulation and reducing ethanol-mediated oxidative stress. J. Agric. Food Chem. 63 (9), 2413–2422. 10.1021/jf502990n 25665731

[B24] Dulloo A. G. Montani J. P. (2015). Pathways from dieting to weight regain, to obesity and to the metabolic syndrome: an overview. Obes. Rev. 16 (Suppl 1), 1–6. 10.1111/obr.12250 25614198

[B25] Engin A. B. (2017). What is lipotoxicity?. Adv. Exp. Med. Biol. 960, 197–220. 10.1007/978-3-319-48382-5_8 28585200

[B26] Fajas L. Schoonjans K. Gelman L. Kim J. B. Najib J. Martin G. (1999). Regulation of peroxisome proliferator-activated receptor gamma expression by adipocyte differentiation and determination factor 1/sterol regulatory element binding protein 1: implications for adipocyte differentiation and metabolism. Mol. Cell. Biol. 19 (8), 5495–5503. 10.1128/mcb.19.8.5495 10409739 PMC84391

[B27] Fan J. S. Liu D. N. Huang G. Xu Z. Z. Jia Y. Zhang H. G. (2012). Panax notoginseng saponins attenuate atherosclerosis via reciprocal regulation of lipid metabolism and inflammation by inducing liver X receptor alpha expression. J. Ethnopharmacol. 142 (3), 732–738. 10.1016/j.jep.2012.05.053 22683903

[B28] Fei H. Li M. Liu W. Sun L. Li N. Cao L. (2016). Potential lipase inhibitors from Chinese medicinal herbs. Pharm. Biol. 54 (12), 2845–2850. 10.1080/13880209.2016.1185635 27267857

[B29] Fernandez-Marcos P. J. Auwerx J. (2011). Regulation of PGC-1α, a nodal regulator of mitochondrial biogenesis. Am. J. Clin. Nutr. 93 (4), 884S–890S. 10.3945/ajcn.110.001917 21289221 PMC3057551

[B30] Fernández-Veledo S. Vázquez-Carballo A. Vila-Bedmar R. Ceperuelo-Mallafré V. Vendrell J. (2013). Role of energy- and nutrient-sensing kinases AMP-activated protein kinase (AMPK) and mammalian target of rapamycin (mTOR) in adipocyte differentiation. IUBMB Life 65 (7), 572–583. 10.1002/iub.1170 23671028

[B31] Filippatos T. D. Derdemezis C. S. Gazi I. F. Nakou E. S. Mikhailidis D. P. Elisaf M. S. (2008). Orlistat-associated adverse effects and drug interactions: a critical review. Drug Saf. 31 (1), 53–65. 10.2165/00002018-200831010-00005 18095746

[B32] Furukawa S. Fujita T. Shimabukuro M. Iwaki M. Yamada Y. Nakajima Y. (2004). Increased oxidative stress in obesity and its impact on metabolic syndrome. J. Clin. Invest. 114 (12), 1752–1761. 10.1172/JCI21625 15599400 PMC535065

[B33] Giordano A. Smorlesi A. Frontini A. Barbatelli G. Cinti S. (2014). White, brown and pink adipocytes: the extraordinary plasticity of the adipose organ. Eur. J. Endocrinol. 170 (5), R159–R171. 10.1530/EJE-13-0945 24468979

[B34] González-Burgos E. Fernández-Moriano C. Lozano R. Iglesias I. Gómez-Serranillos M. P. (2017). Ginsenosides Rd and Re co-treatments improve rotenone-induced oxidative stress and mitochondrial impairment in SH-SY5Y neuroblastoma cells. Food Chem. Toxicol. 109 (Pt 1), 38–47. 10.1016/j.fct.2017.08.013 28843595

[B35] Greenway F. L. Shanahan W. Fain R. Ma T. Rubino D. (2016). Safety and tolerability review of lorcaserin in clinical trials. Clin. Obes. 6 (5), 285–295. 10.1111/cob.12159 27627785

[B36] Gu W. Kim K. A. Kim D. H. (2013). Ginsenoside Rh1 ameliorates high fat diet-induced obesity in mice by inhibiting adipocyte differentiation. Biol. Pharm. Bull. 36 (1), 102–107. 10.1248/bpb.b12-00558 23302642

[B37] Guo X. Sun W. Luo G. Wu L. Xu G. Hou D. (2019). Panax notoginseng saponins alleviate skeletal muscle insulin resistance by regulating the IRS1-PI3K-AKT signaling pathway and GLUT4 expression. FEBS Open Bio 9 (5), 1008–1019. 10.1002/2211-5463.12635 PMC6487711 30945455

[B38] Halpern B. Halpern A. (2015). Safety assessment of FDA-approved (orlistat and lorcaserin) anti-obesity medications. Expert Opin. Drug Saf. 14 (2), 305–315. 10.1517/14740338.2015.994502 25563411

[B39] Halpern B. Mancini M. C. (2017). Safety assessment of combination therapies in the treatment of obesity: focus on naltrexone/bupropion extended release and phentermine-topiramate extended release. Expert Opin. Drug Saf. 16 (1), 27–39. 10.1080/14740338.2017.1247807 27732121

[B40] Han M. Han L. M. Wang Q. S. Bai Z. H. Fang X. L. (2006). [Mechanism of oral absorption of panaxnotoginseng saponins]. Yao Xue Xue Bao 41 (6), 498–505. 16927822

[B41] Hardie D. G. (2011). AMP-activated protein kinase: an energy sensor that regulates all aspects of cell function. Genes Dev. 25 (18), 1895–1908. 10.1101/gad.17420111 21937710 PMC3185962

[B42] Harms M. Seale P. (2013). Brown and beige fat: development, function and therapeutic potential. Nat. Med. 19 (10), 1252–1263. 10.1038/nm.3361 24100998

[B43] Hondares E. Rosell M. Díaz-Delfín J. Olmos Y. Monsalve M. Iglesias R. (2011). Peroxisome proliferator-activated receptor α (PPARα) induces PPARγ coactivator 1α (PGC-1α) gene expression and contributes to thermogenic activation of Brown fat. J. Biol. Chem. 286 (50), 43112–43122. 10.1074/jbc.M111.252775 22033933 PMC3234861

[B44] Hong Y. Lin Y. Si Q. Yang L. Dong W. Gu X. (2019). Ginsenoside Rb2 alleviates obesity by activation of Brown fat and induction of browning of white fat. Front. Endocrinol. (Lausanne) 10, 153. 10.3389/fendo.2019.00153 30930854 PMC6428988

[B45] Hou Y. Gu D. Peng J. Jiang K. Li Z. Shi J. (2020). Ginsenoside Rg1 regulates liver lipid factor metabolism in NAFLD model rats. ACS Omega 5 (19), 10878–10890. 10.1021/acsomega.0c00529 32455208 PMC7241038

[B46] Hu S. Liu T. Wu Y. Yang W. Hu S. Sun Z. (2019). Panax notoginseng saponins suppress lipopolysaccharide-induced barrier disruption and monocyte adhesion on bEnd.3 cells via the opposite modulation of Nrf2 antioxidant and NF-κB inflammatory pathways. Phytother Res. 33 (12), 3163–3176. 10.1002/ptr.6488 31468630

[B47] Hu S. Wu Y. Zhao B. Hu H. Zhu B. Sun Z. (2018). Panax notoginseng saponins protect cerebral microvascular endothelial cells against oxygen-glucose deprivation/reperfusion-induced barrier dysfunction via activation of PI3K/Akt/Nrf2 antioxidant signaling pathway. Molecules 23 (11), 2781. 10.3390/molecules23112781 PMC6278530 30373188

[B48] Huang Y. C. Lin C. Y. Huang S. F. Lin H. C. Chang W. L. Chang T. C. (2010). Effect and mechanism of ginsenosides CK and Rg1 on stimulation of glucose uptake in 3T3-L1 adipocytes. J. Agric. Food Chem. 58 (10), 6039–6047. 10.1021/jf9034755 20441170

[B49] Hwang J. T. Lee M. S. Kim H. J. Sung M. J. Kim H. Y. Kim M. S. (2009). Antiobesity effect of ginsenoside Rg3 involves the AMPK and PPAR-gamma signal pathways. Phytother Res. 23 (2), 262–266. 10.1002/ptr.2606 18844326

[B50] Jia Y. Li Z. Y. Zhang H. G. Li H. B. Liu Y. Li X. H. (2010). Panax notoginseng saponins decrease cholesterol ester via up-regulating ATP-binding cassette transporter A1 in foam cells. J. Ethnopharmacol. 132 (1), 297–302. 10.1016/j.jep.2010.08.033 20727959

[B51] Kaviani M. Keshtkar S. Azarpira N. Hossein Aghdaei M. Geramizadeh B. Karimi M. H. (2019). Cytoprotective effects of ginsenoside Rd on apoptosis-associated cell death in the isolated human pancreatic islets. Excli J. 18, 666–676. 10.17179/excli2019-1698 31611749 PMC6785759

[B52] Kim, E. J. Lee H. I. Chung K. J. Noh Y. H. Ro Y. Koo J. H. (2009). The ginsenoside-Rb2 lowers cholesterol and triacylglycerol levels in 3T3-L1 adipocytes cultured under high cholesterol or fatty acids conditions. BMB Rep. 42 (4), 194–199. 10.5483/bmbrep.2009.42.4.194 19403041

[B53] Kim J. B. Spiegelman B. M. (1996). ADD1/SREBP1 promotes adipocyte differentiation and gene expression linked to fatty acid metabolism. Genes Dev. 10 (9), 1096–1107. 10.1101/gad.10.9.1096 8654925

[B54] Kim K. Nam K. H. Yi S. A. Park J. W. Han J.-W. Lee J. (2020). Ginsenoside Rg3 induces browning of 3T3-L1 adipocytes by activating AMPK signaling. Nutrients 12 (2), 427. 10.3390/nu12020427 PMC7071202 32046061

[B55] Kim, M. Ahn B. Y. Lee J. S. Chung S. S. Lim S. Park S. G. (2009). The ginsenoside Rg3 has a stimulatory effect on insulin signaling in L6 myotubes. Biochem. Biophys. Res. Commun. 389 (1), 70–73. 10.1016/j.bbrc.2009.08.088 19699714

[B56] Koh E. J. Kim K. J. Choi J. Jeon H. J. Seo M. J. Lee B. Y. (2017). Ginsenoside Rg1 suppresses early stage of adipocyte development via activation of C/EBP homologous protein-10 in 3T3-L1 and attenuates fat accumulation in high fat diet-induced obese zebrafish. J. Ginseng Res. 41 (1), 23–30. 10.1016/j.jgr.2015.12.005 28123318 PMC5223064

[B57] Kopelman P. G. (2000). Obesity as a medical problem. Nature 404 (6778), 635–643. 10.1038/35007508 10766250

[B58] Koves T. R. Ussher J. R. Noland R. C. Slentz D. Mosedale M. Ilkayeva O. (2008). Mitochondrial overload and incomplete fatty acid oxidation contribute to skeletal muscle insulin resistance. Cell Metab. 7 (1), 45–56. 10.1016/j.cmet.2007.10.013 18177724

[B59] Kutyavin V. I. Chawla A. (2019). BCL6 regulates brown adipocyte dormancy to maintain thermogenic reserve and fitness. Proc. Natl. Acad. Sci. U. S. A. 116 (34), 17071–17080. 10.1073/pnas.1907308116 31375635 PMC6708354

[B60] Lee, H. S. Lim S.-M. Jung J. I. Kim S. M. Lee J. K. Kim Y. H. (2019). Gynostemma pentaphyllum extract ameliorates high-fat diet-induced obesity in C57BL/6N mice by upregulating SIRT1. Nutrients 11 (10), 2475. 10.3390/nu11102475 PMC6835433 31618980

[B61] Lee J. B. Yoon S. J. Lee S. H. Lee M. S. Jung H. Kim T. D. (2017). Ginsenoside Rg3 ameliorated HFD-induced hepatic steatosis through downregulation of STAT5-PPARγ. J. Endocrinol. 235 (3), 223–235. 10.1530/JOE-17-0233 29042402

[B62] Lee, J. H. Park A. Oh K. J. Lee S. C. Kim W. K. Bae K. H. (2019). The role of adipose tissue mitochondria: regulation of mitochondrial function for the treatment of metabolic diseases. Ijms 20 (19), 4924. 10.3390/ijms20194924 PMC6801758 31590292

[B63] Lee, K. Seo Y. J. Song J. H. Chei S. Lee B. Y. (2019). Ginsenoside Rg1 promotes browning by inducing UCP1 expression and mitochondrial activity in 3T3-L1 and subcutaneous white adipocytes. J. Ginseng Res. 43 (4), 589–599. 10.1016/j.jgr.2018.07.005 31695565 PMC6823768

[B64] Lee O. H. Lee H. H. Kim J. H. Lee B. Y. (2011). Effect of ginsenosides Rg3 and Re on glucose transport in mature 3T3-L1 adipocytes. Phytother Res. 25 (5), 768–773. 10.1002/ptr.3322 21520470

[B65] Lee S. Lee M. S. Kim C. T. Kim I. H. Kim Y. (2012). Ginsenoside Rg3 reduces lipid accumulation with AMP-Activated Protein Kinase (AMPK) activation in HepG2 cells. Int. J. Mol. Sci. 13 (5), 5729–5739. 10.3390/ijms13055729 22754327 PMC3382760

[B66] Lefterova M. I. Lazar M. A. (2009). New developments in adipogenesis. Trends Endocrinol. Metab. 20 (3), 107–114. 10.1016/j.tem.2008.11.005 19269847

[B67] Li J. B. Zhang R. Han X. Piao C. L. (2018). Ginsenoside Rg1 inhibits dietary-induced obesity and improves obesity-related glucose metabolic disorders. Braz. J. Med. Biol. Res. 51 (4), e7139. 10.1590/1414-431X20177139 29513799 PMC5856439

[B68] Lim S. Park J. Um J. Y. (2019). Ginsenoside Rb1 induces beta 3 adrenergic receptor-dependent lipolysis and thermogenesis in 3T3-L1 adipocytes and db/db mice. Front. Pharmacol. 10, 1154. 10.3389/fphar.2019.01154 31680950 PMC6803469

[B69] Lin Y. Hu Y. Hu X. Yang L. Chen X. Li Q. (2020). Ginsenoside Rb2 improves insulin resistance by inhibiting adipocyte pyroptosis. Adipocyte 9 (1), 302–312. 10.1080/21623945.2020.1778826 32580621 PMC7469678

[B70] Liu, C. Feng R. Zou J. Xia F. Wan J.-B. (2019). 20(S)-Protopanaxadiol saponins mainly contribute to the anti-atherogenic effects of panax notoginseng in ApoE deficient mice. Molecules 24 (20), 3723. 10.3390/molecules24203723 PMC6832312 31623159

[B71] Liu, H. Liu M. Jin Z. Yaqoob S. Zheng M. Cai D. (2019). Ginsenoside Rg2 inhibits adipogenesis in 3T3-L1 preadipocytes and suppresses obesity in high-fat-diet-induced obese mice through the AMPK pathway. Food Funct. 10 (6), 3603–3614. 10.1039/c9fo00027e 31161181

[B72] Liu H. Yang J. Du F. Gao X. Ma X. Huang Y. (2009). Absorption and disposition of ginsenosides after oral administration of Panax notoginseng extract to rats. Drug Metab. Dispos. 37 (12), 2290–2298. 10.1124/dmd.109.029819 19786509

[B73] Liu H. Wang J. Liu M. Zhao H. Yaqoob S. Zheng M. (2018). Antiobesity effects of ginsenoside Rg1 on 3T3-L1 preadipocytes and high fat diet-induced obese mice mediated by AMPK. Nutrients 10 (7), 830. 10.3390/nu10070830 PMC6073290 29954059

[B74] Liu, X. W. Lu M. K. Zhong H. T. Wang L. H. Fu Y. P. (2019). Panax notoginseng saponins attenuate myocardial ischemia-reperfusion injury through the HIF-1α/BNIP3 pathway of autophagy. J. Cardiovasc. Pharmacol. 73 (2), 92–99. 10.1097/FJC.0000000000000640 30531436

[B75] Lizcano F. (2019). The beige adipocyte as a therapy for metabolic diseases. Int. J. Mol. Sci. 20 (20). 10.3390/ijms20205058 PMC6834159 31614705

[B76] Masuno H. Kitao T. Okuda H. (1996). Ginsenosides increase secretion of lipoprotein lipase by 3T3-L1 adipocytes. Biosci. Biotechnol. Biochem. 60 (12), 1962–1965. 10.1271/bbb.60.1962 8988629

[B77] Morigny P. Houssier M. Mouisel E. Langin D. (2016). Adipocyte lipolysis and insulin resistance. Biochimie 125, 259–266. 10.1016/j.biochi.2015.10.024 26542285

[B78] Morino K. Petersen K. F. Shulman G. I. (2006). Molecular mechanisms of insulin resistance in humans and their potential links with mitochondrial dysfunction. Diabetes 55 (Suppl. 2), S9–S15. 10.2337/db06-S002 17130651 PMC2995546

[B79] Moseti D. Regassa A. Kim W. K. (2016). Molecular regulation of adipogenesis and potential anti-adipogenic bioactive molecules. Int. J. Mol. Sci. 17 (1), 124. 10.3390/ijms17010124 PMC4730365 26797605

[B80] Mota M. Banini B. A. Cazanave S. C. Sanyal A. J. (2016). Molecular mechanisms of lipotoxicity and glucotoxicity in nonalcoholic fatty liver disease. Metab. Clin. Exp. 65 (8), 1049–1061. 10.1016/j.metabol.2016.02.014 26997538 PMC4931958

[B81] Mottillo E. P. Bloch A. E. Leff T. Granneman J. G. (2012). Lipolytic products activate peroxisome proliferator-activated receptor (PPAR) α and δ in brown adipocytes to match fatty acid oxidation with supply. J. Biol. Chem. 287 (30), 25038–25048. 10.1074/jbc.M112.374041 22685301 PMC3408177

[B82] Mozzini C. Garbin U. Stranieri C. Pasini A. Solani E. Tinelli I. A. (2015). Endoplasmic reticulum stress and Nrf2 repression in circulating cells of type 2 diabetic patients without the recommended glycemic goals. Free Radic. Res. 49 (3), 244–252. 10.3109/10715762.2014.997229 25511473

[B83] Mu Q. Fang X. Li X. Zhao D. Mo F. Jiang G. (2015). Ginsenoside Rb1 promotes browning through regulation of PPARγ in 3T3-L1 adipocytes. Biochem. Biophys. Res. Commun. 466 (3), 530–535. 10.1016/j.bbrc.2015.09.064 26381176

[B84] Ohno H. Shinoda K. Spiegelman B. M. Kajimura S. (2012). PPARγ agonists induce a white-to-brown fat conversion through stabilization of PRDM16 protein. Cell Metab. 15 (3), 395–404. 10.1016/j.cmet.2012.01.019 22405074 PMC3410936

[B85] Park, H. J. Kim J. H. Shim I. (2019). Anti-obesity effects of ginsenosides in high-fat diet-fed rats. Chin. J. Integr. Med. 25 (12), 895–901. 10.1007/s11655-019-3200-x 31144161

[B86] Park M. W. Ha J. Chung S. H. (2008). 20(S)-ginsenoside Rg3 enhances glucose-stimulated insulin secretion and activates AMPK. Biol. Pharm. Bull. 31 (4), 748–751. 10.1248/bpb.31.748 18379076

[B87] Park, S. J. Park M. Sharma A. Kim K. Lee H. J. (2019). Black ginseng and ginsenoside Rb1 promote browning by inducing UCP1 expression in 3T3-L1 and primary white adipocytes. Nutrients 11 (11), 2747. 10.3390/nu11112747 PMC6893667 31726767

[B88] Peng S. Wang Y. Zhou Y. Ma T. Wang Y. Li J. (2019). Rare ginsenosides ameliorate lipid overload-induced myocardial insulin resistance via modulating metabolic flexibility. Phytomedicine 58, 152745. 10.1016/j.phymed.2018.11.006 31005715

[B89] Qiang L. Wang L. Kon N. Zhao W. Lee S. Zhang Y. (2012). Brown remodeling of white adipose tissue by SirT1-dependent deacetylation of Pparγ. Cell 150 (3), 620–632. 10.1016/j.cell.2012.06.027 22863012 PMC3413172

[B90] Qiao Y. J. Shang J. H. Wang D. Zhu H. T. Yang C. R. Zhang Y. J. (2018). Research of panax spp. in kunming institute of botany, CAS. Nat. Prod. Bioprospect 8 (4), 245–263. 10.1007/s13659-018-0176-8 29980943 PMC6102176

[B91] Reeds D. N. Patterson B. W. Okunade A. Holloszy J. O. Polonsky K. S. Klein S. (2011). Ginseng and ginsenoside Re do not improve β-cell function or insulin sensitivity in overweight and obese subjects with impaired glucose tolerance or diabetes. Diabetes Care 34 (5), 1071–1076. 10.2337/dc10-2299 21411505 PMC3114517

[B92] Reusch J. E. Colton L. A. Klemm D. J. (2000). CREB activation induces adipogenesis in 3T3-L1 cells. Mol. Cell. Biol. 20 (3), 1008–1020. 10.1128/mcb.20.3.1008-1020.2000 10629058 PMC85218

[B93] Richardson D. K. Kashyap S. Bajaj M. Cusi K. Mandarino S. J. Finlayson J. (2005). Lipid infusion decreases the expression of nuclear encoded mitochondrial genes and increases the expression of extracellular matrix genes in human skeletal muscle. J. Biol. Chem. 280 (11), 10290–10297. 10.1074/jbc.M408985200 15598661

[B94] Rosen E. D. Spiegelman B. M. (2014). What we talk about when we talk about fat. Cell 156 (1-2), 20–44. 10.1016/j.cell.2013.12.012 24439368 PMC3934003

[B95] Rosen E. D. Walkey C. J. Puigserver P. Spiegelman B. M. (2000). Transcriptional regulation of adipogenesis. Genes Dev. 14 (11), 1293–1307. 10.1002/cphy.c160022 10837022

[B96] Rupasinghe H. P. Sekhon-Loodu S. Mantso T. Panayiotidis M. I. (2016). Phytochemicals in regulating fatty acid β-oxidation: potential underlying mechanisms and their involvement in obesity and weight loss. Pharmacol. Ther. 165, 153–163. 10.1016/j.pharmthera.2016.06.005 27288729

[B97] Sartipy P. Loskutoff D. J. (2003). Monocyte chemoattractant protein 1 in obesity and insulin resistance. Proc. Natl. Acad. Sci. USA 100 (12), 7265–7270. 10.1073/pnas.1133870100 12756299 PMC165864

[B98] Semirale A. A. Zhang X. W. Wiren K. M. (2011). Body composition changes and inhibition of fat development *in vivo* implicates androgen in regulation of stem cell lineage allocation. J. Cell. Biochem. 112 (7), 1773–1786. 10.1002/jcb.23098 21381083 PMC3111903

[B99] Shang W. B. Guo C. Zhao J. Yu X. Z. Zhang H. (2014). Ginsenoside Rb1 upregulates expressions of GLUTs to promote glucose consumption in adiopcytes. Zhongguo Zhong Yao Za Zhi 39 (22), 4448–4452. 25850283

[B100] Shen L. Xiong Y. Wang D. Q. Howles P. Basford J. E. Wang J. (2013). Ginsenoside Rb1 reduces fatty liver by activating AMP-activated protein kinase in obese rats. J. Lipid Res. 54 (5), 1430–1438. 10.1194/jlr.M035907 23434611 PMC3622335

[B101] Siebenhofer A. Jeitler K. Horvath K. Berghold A. Posch N. Meschik J. (2016). Long-term effects of weight-reducing drugs in people with hypertension. Cochrane Database Syst. Rev. 3, CD007654. 10.1002/14651858.CD007654.pub4 26934640

[B102] Siraj F. M. SathishKumar N. Kim Y. J. Kim S. Y. Yang D. C. (2015). Ginsenoside F2 possesses anti-obesity activity via binding with PPARγ and inhibiting adipocyte differentiation in the 3T3-L1 cell line. J. Enzyme Inhib. Med. Chem. 30 (1), 9–14. 10.3109/14756366.2013.871006 24666293

[B103] Smith U. Kahn B. B. (2016). Adipose tissue regulates insulin sensitivity: role of adipogenesis, de novo lipogenesis and novel lipids. J. Intern. Med. 280 (5), 465–475. 10.1111/joim.12540 27699898 PMC5218584

[B104] Sparks L. M. Xie H. Koza R. A. Mynatt R. Hulver M. W. Bray G. A. (2005). A high-fat diet coordinately downregulates genes required for mitochondrial oxidative phosphorylation in skeletal muscle. Diabetes 54 (7), 1926–1933. 10.2337/diabetes.54.7.1926 15983191

[B105] Su S. Guntur A. R. Nguyen D. C. Fakory S. S. Doucette C. C. Leech C. (2018). A renewable source of human beige adipocytes for development of therapies to treat metabolic syndrome. Cell Rep 25 (11), 3215–3219.e9. 10.1016/j.celrep.2018.11.037 30540952 PMC6375695

[B106] van Dam A. D. Kooijman S. Schilperoort M. Rensen P. C. Boon M. R. (2015). Regulation of brown fat by AMP-activated protein kinase. Trends Mol. Med. 21 (9), 571–579. 10.1016/j.molmed.2015.07.003 26271143

[B107] Villalobos-Labra R. Subiabre M. Toledo F. Pardo F. Sobrevia L. (2019). Endoplasmic reticulum stress and development of insulin resistance in adipose, skeletal, liver, and foetoplacental tissue in diabesity. Mol. Aspects Med. 66, 49–61. 10.1016/j.mam.2018.11.001 30472165

[B108] Wang Q. Mu R. F. Liu X. Zhou H. M. Xu Y. H. Qin W. Y. (2020). Steaming changes the composition of saponins of panax notoginseng (burk.) F.H. Chen that function in treatment of hyperlipidemia and obesity. J. Agric. Food Chem. 68 (17), 4865–4875. 10.1021/acs.jafc.0c00746 32306731

[B109] Wang W. Hao Y. Li F. (2019). Notoginsenoside R1 alleviates high glucose-evoked damage in RSC96 cells through down-regulation of miR-503. Artif. Cell. Nanomed. Biotechnol. 47 (1), 3947–3954. 10.1080/21691401.2019.1671434 31581849

[B110] Wang Y. Liang X. Chen Y. Zhao X. (2016). Screening SIRT1 activators from medicinal plants as bioactive compounds against oxidative damage in mitochondrial function. Oxid Med. Cell. Longev. 2016, 4206392. 10.1155/2016/4206392 26981165 PMC4766345

[B111] Wang Y. X. Lee C. H. Tiep S. Yu R. T. Ham J. Kang H. (2003). Peroxisome-proliferator-activated receptor delta activates fat metabolism to prevent obesity. Cell 113 (2), 159–170. 10.1016/s0092-8674(03)00269-1 12705865

[B112] Weisberg S. P. McCann D. Desai M. Rosenbaum M. Leibel R. L. Ferrante A. W. Jr. (2003). Obesity is associated with macrophage accumulation in adipose tissue. J. Clin. Invest. 112 (12), 1796–1808. 10.1172/JCI19246 14679176 PMC296995

[B113] Wu Y. Huang X. F. Bell C. Yu Y. (2018). Ginsenoside Rb1 improves leptin sensitivity in the prefrontal cortex in obese mice. CNS Neurosci. Ther. 24 (2), 98–107. 10.1111/cns.12776 29130652 PMC6490040

[B114] Wu Y. Yu Y. Szabo A. Han M. Huang X. F. (2014). Central inflammation and leptin resistance are attenuated by ginsenoside Rb1 treatment in obese mice fed a high-fat diet. PLoS One 9 (3), e92618. 10.1371/journal.pone.0092618 24675731 PMC3968027

[B115] Wu Z. Rosen E. D. Brun R. Hauser S. Adelmant G. Troy A. E. (1999). Cross-regulation of C/EBP alpha and PPAR gamma controls the transcriptional pathway of adipogenesis and insulin sensitivity. Mol. Cell. 3 (2), 151–158. 10.1016/s1097-2765(00)80306-8 10078198

[B116] Xiang H. Liu Y. Zhang B. Huang J. Li Y. Yang B. (2011). The antidepressant effects and mechanism of action of total saponins from the caudexes and leaves of Panax notoginseng in animal models of depression. Phytomedicine 18 (8-9), 731–738. 10.1016/j.phymed.2010.11.014 21273053

[B117] Xie Z. Loi Truong T. Zhang P. Xu F. Xu X. Li P. (2015). Dan-Qi prescription ameliorates insulin resistance through overall corrective regulation of glucose and fat metabolism. J. Ethnopharmacol. 172, 70–79. 10.1016/j.jep.2015.05.041 26087232

[B118] Xing W. Yang L. Peng Y. Wang Q. Gao M. Yang M. (2017). Ginsenoside Rg3 attenuates sepsis-induced injury and mitochondrial dysfunction in liver via AMPK-mediated autophagy flux. Biosci. Rep. 37 (4). 10.1042/BSR20170934 PMC5577177 28779013

[B119] Xiong Y. Shen L. Liu K. J. Tso P. Xiong Y. Wang G. (2010). Antiobesity and antihyperglycemic effects of ginsenoside Rb1 in rats. Diabetes 59 (10), 2505–2512. 10.2337/db10-0315 20682695 PMC3279544

[B120] Xu Y. Wang N. Tan H.-Y. Li S. Zhang C. Zhang Z. (2020). Panax notoginseng saponins modulate the gut microbiota to promote thermogenesis and beige adipocyte reconstruction via leptin-mediated AMPKα/STAT3 signaling in diet-induced obesity. Theranostics 10 (24), 11302–11323. 10.7150/thno.47746 33042284 PMC7532683

[B121] Yang C. Y. Xie Z. G. Cheng W. B. Jiang X. Chen Z. H. (2009). [Effects of Panax notoginseng saponins on anti-hyperglycemic, anti-obese and prevention from kidney pathological changes in KK-Ay mice]. Zhong Yao Cai 32 (10), 1571–1576. 20112724

[B122] Yang J. W. Kim S. S. (2015). Ginsenoside Rc promotes anti-adipogenic activity on 3T3-L1 adipocytes by down-regulating C/EBPα and PPARγ. Molecules 20 (1), 1293–1303. 10.3390/molecules20011293 25594343 PMC6272142

[B123] Yao L. Han Z. Zhao G. Xiao Y. Zhou X. Dai R. (2020). Ginsenoside Rd ameliorates high fat diet‐induced obesity by enhancing adaptive thermogenesis in a cAMP‐dependent manner. Obesity 28 (4), 783–792. 10.1002/oby.22761 32144882

[B124] Yu X. Ye L. Zhang H. Zhao J. Wang G. Guo C. (2015). Ginsenoside Rb1 ameliorates liver fat accumulation by upregulating perilipin expression in adipose tissue of db/db obese mice. J. Ginseng Res. 39 (3), 199–205. 10.1016/j.jgr.2014.11.004 26199550 PMC4506369

[B125] Yuan H. D. Kim J. T. Kim S. H. Chung S. H. (2012). Ginseng and diabetes: the evidences from *in vitro*, animal and human studies. J. Ginseng Res. 36 (1), 27–39. 10.5142/jgr.2012.36.1.27 23717101 PMC3659569

[B126] Zhang B. Berger J. Hu E. Szalkowski D. White-Carrington S. Spiegelman B. M. (1996). Negative regulation of peroxisome proliferator-activated receptor-gamma gene expression contributes to the antiadipogenic effects of tumor necrosis factor-alpha. Mol. Endocrinol. 10 (11), 1457–1466. 10.1210/mend.10.11.8923470 8923470

[B127] Zhang H. Chen Z. Zhong Z. Gong W. Li J. (2018). Total saponins from the leaves of Panax notoginseng inhibit depression on mouse chronic unpredictable mild stress model by regulating circRNA expression. Brain Behav. 8 (11), e01127. 10.1002/brb3.1127 30298999 PMC6236231

[B128] Zhang L. Zhang L. Wang X. Si H. (2017). Anti-adipogenic effects and mechanisms of ginsenoside Rg3 in pre-adipocytes and obese mice. Front. Pharmacol. 8, 113. 10.3389/fphar.2017.00113 28337143 PMC5340763

[B129] Zhou Z. Wang J. Song Y. He Y. Zhang C. Liu C. (2018). Panax notoginseng saponins attenuate cardiomyocyte apoptosis through mitochondrial pathway in natural aging rats. Phytother Res. 32 (2), 243–250. 10.1002/ptr.5961 29130614

